# Comparative Performance Evaluation of FilmArray BioFire RP2.1 and MAScIR 2.0 Assays for SARS-CoV-2 Detection

**DOI:** 10.1155/2022/4510900

**Published:** 2022-06-01

**Authors:** Sophia Tazi, Hakima Kabbaj, Jalila Zirar, Amal Zouaki, Ghizlane El Amin, Othman El Himeur, Myriam Seffar

**Affiliations:** ^1^Mohamed V University, Faculty of Medicine and Pharmacy, Rabat, Morocco; ^2^Ibn Sina University Hospital Center, Central Laboratory of Virology, Rabat, Morocco; ^3^Ibn Sina University Hospital Center, Central Laboratory of Bacteriology, Rabat, Morocco

## Abstract

**Background:**

RT-PCR is the gold standard for COVID-19 diagnosis, but the lack of standardization of assays, whose diagnostic performance may widely vary, complicates the interpretation of the discrepancies that may be encountered. *Study design*. We conducted a retrospective study over a ten-month period at the Central Laboratory of Virology of Ibn Sina University Hospital of Rabat. We included nasopharyngeal swabs, positive and negative for SARS-CoV-2 on FilmArray BioFire® Respiratory Panel 2.1 Plus, which were subjected to our laboratory's reference test, MAScIR SARS-CoV-2 M kit 2.0, initially or after a freeze-thaw cycle. The results were compared, and each discrepant sample with sufficient volume underwent the third test, using ARGENE® SARS-CoV-2 R-GENE kit.

**Results:**

Of 80 SARS-CoV-2 negative samples on FilmArray, there were no discordant results, whereas of 80 SARS-CoV-2 positive samples on FilmArray, 21 had discordant results on MAScIR, and only 11 could be tested on ARGENE, revealing positive results in 6 cases. 12.7% and 76.5% correspond to the discordance rates for MAScIR (with one or both targets detected on FilmArray), while 14.3% and 100% correspond to those of ARGENE. As the estimated sensitivity and specificity of FilmArray, compared with MAScIR, were 100% and 79.2%, respectively, its lower limit of detection, and ARGENE assay results, made it difficult to distinguish between false positives on FilmArray and false negatives on MAScIR without further investigations.

**Conclusion:**

The implementation of a new assay in our laboratory revealed discrepancies suggesting a lack of sensitivity of our laboratory's reference test, leading us consequently to retain the SARS-CoV-2 positive result of these discordant samples on FilmArray, regardless of the detection of one or both targets. Our study, which is, to our knowledge, the first comparing FilmArray RP2.1 and MAScIR 2.0 assays for SARS-CoV-2 detection, highlights the urgent need to standardize RT-PCR assays for COVID-19 diagnosis.

## 1. Introduction

In late December 2019, a cluster of pneumonia cases of unknown etiology was reported in Wuhan, China. Ultimately, a novel *betacoronavirus* named severe acute respiratory syndrome coronavirus 2 (SARS-CoV-2) was identified as the causative agent and subsequently isolated and sequenced. It is a highly pathogenic virus, causing a disease, referred to as COVID-19, which has been declared a global pandemic by the World Health Organization (WHO) in March 2020. SARS-CoV-2 belongs to the *Coronaviridae* family of enveloped positive-stranded RNA viruses that exhibit the largest RNA genome of all known viruses. Two-thirds of its genome encodes for nonstructural proteins, including the RNA-dependent RNA polymerase (RdRp) which is responsible for viral RNA replication and transcription, while the remaining one-third encodes for four main structural proteins, namely: Spike (S), Envelope (E), Membrane (M), and Nucleocapsid (N) proteins [1, 2].

Mass testing has been recommended from the early days of the pandemic by the WHO for the surveillance and control of the spread of the disease, which required the rapid development of numerous diagnostic assays to increase testing capabilities, including RT-PCR assays, which represent the gold standard for COVID-19 diagnosis. Thus, many kits have been developed which have not been formally approved but received emergency use authorizations from the regulatory agencies. However, since these tests are not standardized, their comparison may reveal wide disparities in diagnostic performance, which can severely compromise the effectiveness of disease control programs.

In our laboratory, the recent implementation of a new nested multiplex PCR assay, FilmArray BioFire® Respiratory Panel 2.1 Plus (RP2.1), led us to conduct a comparative study between the latter and our laboratory's reference test, MAScIR SARS-CoV-2 M kit 2.0, which aimed to evaluate the performance of the first one for SARS-CoV-2 detection, in comparison with the second one. The discrepant samples were subsequently tested using ARGENE® SARS-CoV-2 R-GENE kit.

## 2. Materials and Methods

### 2.1. Study Design and Clinical Specimens

We conducted a retrospective study over a ten-month period, from January 1 to October 31, 2021, at the Central Laboratory of Virology (LCV) of Ibn Sina University Hospital of Rabat.

Our study included all nasopharyngeal swabs collected from the various departments of the Hospital and sent to the LCV in viral transport media or in sterile saline water, which were positive for SARS-CoV-2 on FilmArray RP2.1. Of these samples, the samples that were not initially tested on MAScIR 2.0 were removed from storage at −70°C, underwent a freeze-thaw cycle, and were subjected to the latter test. At this stage, we excluded five samples due to their insufficient volume. Secondly, we included an equal number of SARS-CoV-2 negative samples on FilmArray RP2.1, which required the extraction of data from the laboratory information system (eLabs, ENOVA Research and Technology), collected during the study period, which was exported to an Excel document, and then performed a random selection of 80 samples tested simultaneously on FilmArray RP2.1 and MAScIR 2.0. Finally, each sample with discordant results and sufficient volume underwent an ARGENE R-GENE RT-PCR.

### 2.2. FilmArray BioFire® RP2.1 Plus PCR

It is a multiplex nested PCR, performed in a closed and autonomous system, allowing the simultaneous detection of 4 bacteria and 19 viruses (Table 1), including SARS-CoV-2, whose S (Spike) and M (Membrane) genes are targeted.

According to the manufacturer's instructions, 300 *μ*L of sample is mixed with sample buffer and injected into a test pouch containing all necessary reagents for nucleic extraction, amplification, and target detection. Each run includes two controls. The software automatically interprets the endpoint melting curve data to provide a qualitative result for each target. A microorganism is reported as detected if at least one of its corresponding assays is positive [3].

### 2.3. MAScIR SARS-CoV-2 M Kit 2.0 RT-PCR

It is a triplex real-time RT-PCR, using TaqMan technology, targeting SARS-CoV-2 RdRp (RNA-dependent RNA polymerase) and S genes, and the human ribonuclease P (RNase P) gene as an internal control.

RNA was extracted from 200 or 300 *μ*L of the sample, mixed with or without protein kinase, on one of the following platforms: GenePure Pro® (BioEr®), BigFish®, Molarray®, and Maxwell® RSC Instrument (Promega®). The last one performs on single cartridges while the other three use 16-well plates. Subsequently, 6.5 *μ*L of eluted nucleic acid was added to 3.5 *μ*L of the reaction mixture. Amplification was then performed on one of these thermocyclers: ABI 7500 FAST Real-Time PCR System (Applied Biosystems™), QuantStudio™ 5 DX Real-Time PCR System Applied Biosystems™, and Exicycler™ 96 Real-Time Quantitative Thermal Block (Bioneer™), according to the following protocol: 5 minutes at 50°C for reverse transcription, 20 seconds at 95°C for activation, followed by 40 cycles of denaturation at 95°C for 3 seconds, and hybridization/elongation at 60°C for 30 seconds [4]. In each 96-well microplate, three controls were included: one positive and two negatives, of which an extraction negative control.

The semi-quantitative interpretation of the results was based on the manufacturer's instructions, as well as the SFM (French Society of Microbiology) recommendations for this test [5]. An internal control Ct value ≤35 monitors the absence of inhibition and the stability of thawed samples. A sample is positive when its lowest Ct value is ≤30 and low positive when the latter is 30 < Ct ≤ 37. Low positive samples without previous RT-PCR results were retested to rule out sample contamination.

### 2.4. ARGENE® SARS-CoV-2 R-GENE RT-PCR

It is a triplex real-time RT-PCR, using TaqMan technology, targeting SARS-CoV-2 N (Nucleocapsid) and RdRp genes, and an exogenous internal control. This assay can be completed, in equivocal cases, by an optional RT-PCR2 targeting SARSCoV-2 E (Envelope) gene. The latter assay was not required for any of our samples.

About 200 *μ*L of the sample was mixed with 10 *μ*L of internal control prior to the extraction, performed on BigFish®. Subsequently, 10 *μ*L of eluted nucleic acid was added to 15 *μ*L of the reaction mixture. Amplification was then performed on QuantStudio™ 5 DX Real-Time PCR System Applied Biosystems™, according to the following protocol: 5 minutes at 50°C for reverse transcription, 15 minutes at 95°C for activation, followed by 45 cycles of denaturation at 95°C for 10 seconds, hybridization at 60°C for 40 seconds, and elongation at 72°C for 25 seconds. Two controls were included in the microplate: a positive one and a negative extraction one.

The absence of inhibition is monitored by a difference in internal control Ct values ≤3 between the sample and the negative control. According to the manufacturer's instructions, a sample is positive when the N gene Ct value is <40, or when the RdRp gene is detected, whatever its Ct value is. Moreover, according to the SFM recommendations for this assay, a sample is positive when its lowest Ct value is ≤34 and low positive when the latter is >34 [5, 6].

## 3. Results

### 3.1. Characteristics of the Study Population

The 80 positive samples for SARS-CoV-2 on FilmArray RP2.1 were collected from 75 patients, 4 of whom had 2 samples each. The mean age of these patients was 45.2 years and their sex ratio was 1.03. The mean time from symptom onset to swab collection was 6.1 days. Dyspnea, fever, and cough were the most common symptoms. The last sample was a national quality control (NQC), included in a MAScIR 2.0 RT-PCR evaluation program, which was subsequently tested on FilmArray RP2.1.

The 80 negative samples for SARS-CoV-2 on FilmArray RP2.1 were collected from 80 patients, whose mean age was 10.2 years and their sex ratio was 1.58.

### 3.2. Evaluation of FilmArray RP2.1 Performance for SARS-CoV-2 Detection

During the ten-month study period, 595 PCRs were performed on FilmArray RP2.1, of which 85 were positive for SARS-CoV-2, representing a prevalence of 14.3%.

The 80 positive samples included in our study comprised 63 samples (78.8%) with both genes detected, 10 samples with only the S gene detected (12.5%), and 7 samples with only the M gene detected (8.7%).

#### 3.2.1. Comparison with MAScIR 2.0 Assay Results

**Figure 1:**
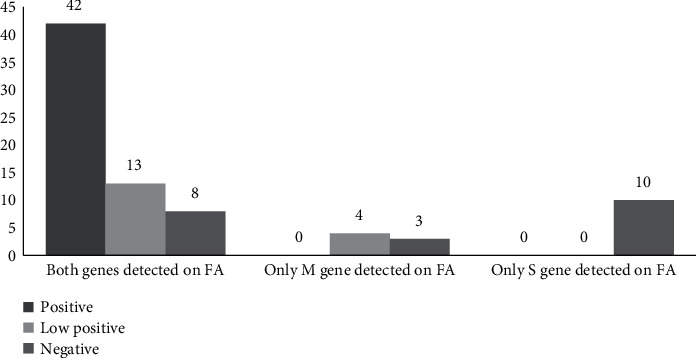
MAScIR 2.0 assay results of the 80 SARS-CoV-2 positive samples on FilmArray RP2.1. FA: FilmArray RP2.1; M: membrane; S: spike.

**Figure 2:**
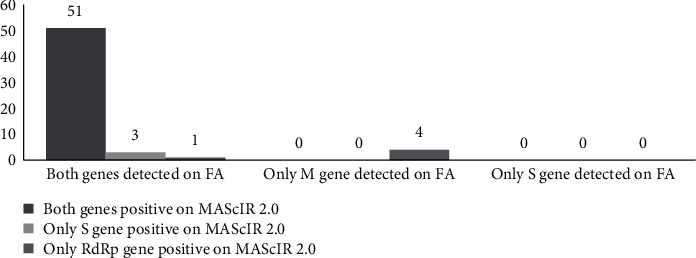
Distribution of the 59 concordant samples according to their positive targets on FilmArray RP2.1 and MAScIR 2.0. FA: FilmArray RP2.1; M: membrane; RdRp: RNA-dependent RNA polymerase; S: spike.

Among the 80 positive samples for SARS-CoV-2 on FilmArray RP2.1, 59 (73.8%) had a concordant result on MAScIR 2.0 (Table 2). These included 42 positive samples, unanimously positive for both S and RdRp genes, and 17 low positive samples, 9 of which were positive for both targets, and 8 of which were positive for a single target with a Ct value ≥35 (Figures 1 and 2). Among the 80 negative samples for SARS-CoV-2 on FilmArray RP2.1, there were no discordant results (Table 2).

Thus, compared with our laboratory's reference test, the sensitivity and specificity of FilmArray RP2.1 were 100% and 79.2%, respectively, and its positive and negative predictive values were 73.8% and 100%, respectively.

The discordance rates ranged from 12.7% when both targets were detected on FilmArray RP2.1, to 42.9% and 100% when only M or S gene was detected, respectively. The 21 discrepant specimens included 8 samples (38%) with both genes detected on FilmArray RP2.1, 3 samples (14%) with only the M gene detected, and 10 samples (48%) with only the S gene detected. Five discrepant specimens were collected from patients who had another sample tested on either assay, a few days apart from the one included in our series (Table 3).

The S gene, only target common to both assays, was unanimously negative on MAScIR 2.0 when negative on FilmArray RP2.1. It was detected on MAScIR 2.0 with a Ct value above the positivity threshold (Ct = 38) in seven samples, two low positives, and five negatives, only one of which was negative for the S gene on FilmArray RP2.1.

#### 3.2.2. Comparison with ARGENE Assay Results

Only 11 discrepant samples could be tested, due to the insufficient volume of the remaining 10 samples. Six samples, with both targets detected on FilmArray RP2.1, had a concordant result on ARGENE, including five positive samples, one of which was only positive for the N gene, and one low positive sample. Five samples were negative on ARGENE, four of which had a single target detected on FilmArray RP2.1 (S in three cases and M in one case).

Thus, the discordance rates ranged from 14.3% when both targets were detected on FilmArray RP2.1, to 100% when only one was detected.

### 3.3. Detection of Other Respiratory Pathogens on FilmArray RP2.1

**Figure 3:**
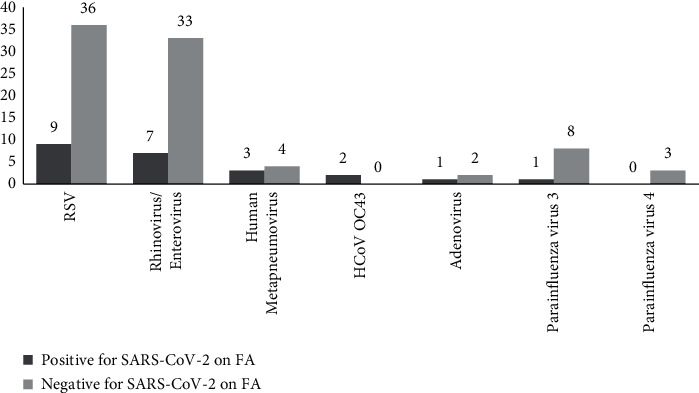
Prevalence of other respiratory viruses detected among SARS-CoV-2 positive and negative samples on FilmArray RP2.1. FA: FilmArray RP2.1; HCoV: human coronavirus; RSV: respiratory syncytial virus.

Among the 80 positive samples for SARS-CoV-2, other viruses were detected in 19 samples, including 4 in which 2 other viruses were identified. Among the 80 negative samples for SARS-CoV-2, 66 tested positive for 1 (72.7%), 2 (24.3%), or 3 (3%) other viruses on the panel. In both cases, respiratory syncytial virus predominated, followed by rhinovirus/enterovirus (Figure 3). Furthermore, no bacterial organisms were detected in our series.

## 4. Discussion

Nucleic acid amplification tests (NAATs), such as RT-PCR, are recognized by the World Health Organization (WHO) and the Centers for Disease Control and Prevention (CDC) as the gold standard for the diagnosis of COVID-19, due to their high sensitivity, specificity, and reproducibility [1, 2]. However, many factors, mostly inherent to the preanalytical phase, can affect their sensitivity. For example, in the study by Kucirka et al., the false-negative rate was 38% on the day of symptom onset and 20% 3 days later. Other studies reported false-negative rates ranging from 1% to 30% [7–10].

In our study, we compared two PCR assays, based on two different principles: FilmArray BioFire® RP2.1 Plus and MAScIR 2.0. The semiquantitative interpretation of the results of the latter was based on the report of its evaluation by the French National Reference Centers (CNR), which established Ct values defining different categories of viral shedding for each assay tested, to cope with the lack of standardization of RT-PCR results [5]. The specificity and positive predictive value of FilmArray RP2.1, both below 80% in our study, were much lower than those estimated in other series (Table 4).

### 4.1. Possible Causes of Discrepancies

Numerous factors may explain the discordant results identified in our series, including:

#### 4.1.1. Variation in Analytical Sensitivity

According to the manufacturers' evaluation reports, the limits of detection (LoD) of FilmArray RP2.1, MAScIR 2.0, and ARGENE are 160, 500, and 380 copies/mL of transport media, respectively [3, 4, 6]. In other studies, the LoD of FilmArray RP2.1 was estimated to be 250 and 302 copies/mL [14, 15]. These data tend to suggest a greater sensitivity of FilmArray RP2.1; however, this cannot be firmly stated as there is no standard material available to determine LoD values. Moreover, these may be reported in different units, making comparisons difficult [1, 16]. The Food and Drug Administration (FDA) and the Coronavirus Standards Working Group (CSWG) have undertaken efforts to address these issues [17–19].

In our study, several arguments plead in favor of a lower sensitivity of MAScIR 2.0, including the following:

- The positive result of 6 (54.5%) of the 11 samples tested on ARGENE, whose LoD value is intermediate between those of our two assays.
- The amplification of the S gene on MAScIR 2.0 in five discordant cases, with Ct values above the positivity threshold.
- The presence, in two discrepant cases (no. 36 and 82—Table 3), of anterior positive results on MAScIR 2.0, reflecting, a priori, the ability of FilmArray RP2.1 to detect a prolonged viral shedding, as reported by Hirotsu et al. [20].

While the analytical impact of the difference in LoD values is not negligible, since each 10-fold increase in LoD is expected to increase the false-negative rate by 13% [16], its clinical impact remains to be defined. Indeed, low positive samples are often collected in the late course of infection, when infectivity is not proven [5, 12, 21–23]. The probability of virus culture isolation has been estimated to be 8% when the Ct value is above 35 [21]. However, the presence of a single low positive sample among the six confirmed positive on ARGENE suggests a lack of sensitivity of our laboratory's reference assay, yet to be confirmed by further investigations.

#### 4.1.2. Variation in Targets

Many authors report a higher sensitivity of assays targeting the E gene and higher specificity of those targeting the ORF1ab, S and N genes, which are nevertheless more prone to mutation [15, 24–27]. Assays' performance must therefore be evaluated in parallel with the monitoring of SARS-CoV-2 genomic evolution, carried out by a WHO working group which reports a relatively low level of SARS-CoV-2 mutations, thanks to its proofreading activity. To date, five variants of concern (VOCs) have been identified, the most recent of which, named Omicron, is characterized by a high transmissibility [28]. FilmArray RP2.1 performance for the detection of the Alpha variant has been evaluated by Jian et al. who reported no false negative results in their series [14]. In our study, some samples tested on MAScIR 2.0 were sequenced and found to be positive for Delta or Omicron variants, confirming that the performance of this assay is not influenced by these mutations.

The kinetics of SARS-CoV-2 infection also seems to influence the positivity of the different targets, as shown in the study by Reina and Suarez [29]. All these arguments underlie the recommendation by the WHO and the French National Authority for Health (HAS) to use RT-PCR assays with at least two independent targets on the SARS-CoV-2 genome [7, 30].

In our study, the discordance rates increased from 12.7% and 14.3% when both targets were detected on FilmArray RP2.1, to 76.5% and 100% when only one was detected, for MAScIR 2.0 and ARGENE, respectively. Given that the three assays' targets are different, with none being common to ARGENE and FilmArray RP2.1 in particular, these rates are most likely due to the targets' kinetic evolution during the SARS-CoV-2 infection, and the detection of a single target on FilmArray RP2.1 would tend to suggest a low positivity of the sample.

#### 4.1.3. Cross-Reactivity

In our series, only one other coronavirus, the HCoV OC43, belonging to the same genus *Betacoronavirus* as SARS-CoV-2, was detected in two discrepant samples (no. 67 and 79—Table 3). A cross-reaction between these two viruses on FilmArray RP2.1 can be suspected, especially in the second case, in which only HCoV OC43 was detected in a second sample collected 2 days later; although contamination of the first sample by SARS-CoV-2 cannot be ruled out. However, no similar cases were reported in the aforementioned series (Table 4) [11–14]. Nevertheless, a cross-reaction with another coronavirus, the HCoV NL63, was suggested by Otsuka et al. who reported in their observation the single detection of SARS-CoV-2 N1 gene on two different assays, Ampdirect™ 2019-nCoV and SARS-CoV-2 Detection Kit. A HCoV NL63 infection was, a posteriori, diagnosed, based on the result of FilmArray RP2.1, which was furthermore negative for SARS-CoV-2. The authors ultimately ruled out the cross-reaction theory and concluded that this case was false positive by instrument or human error, possibly due to sample contamination [31]. In our second case (no. 79), the latter hypothesis, supported by the two negative results on MAScIR 2.0, seems more likely to explain the discrepancy.

#### 4.1.4. Other Possible Causes

In our study, other factors may have interfered with the test results, including:

- deterioration of the samples before the comparative tests.
- poor performance of one of the extractors, the impact of which could not be individually assessed.

### 4.2. Detection of Other Viruses on FilmArray RP2.1

In our study, other pathogens were identified in nearly 24% of the 80 SARS-CoV-2 positive samples on FilmArray RP2.1 and in 11.8% of the 59 samples confirmed positive on MAScIR 2.0. Both rates are much higher than those reported in other series, in which the estimated rates were around 3% [32–34]. Since we did not have access to Ct values on FilmArray RP2.1, we cannot state whether these were true coinfections, sequential infections, or contaminations.

### 4.3. Study Limitations

Our results are subject to inherent limitations which complicate the interpretation of some identified discrepancies, including the following:

- The monocentric and retrospective nature of the study
- The lack of clinical and radiological data provided by clinicians
- The relatively small sample size and the insufficient volume of some specimens
- The variation in extractors over the study period, as well as in thermocyclers for MAScIR 2.0 RT-PCR, the impact of which could not be assessed
- The absence of validation of ARGENE assay in our laboratory prior to its use

## 5. Conclusion

Our study compared the results of 160 samples tested on our two PCR assays and identified 21 discrepant specimens, all negative on our laboratory's reference test, MAScIR 2.0, whose lower analytical sensitivity tends to suggest that these discordant results are most likely false negative ones on this assay. This is supported by the positive results of half of the discrepant samples tested on ARGENE, whose LoD is intermediate between those of our two tests.

The discordance rate was significantly higher when a single target was detected on FilmArray RP2.1, which might suggest that most of our discrepant samples were low positive ones; although this cannot be confirmed without having access to Ct values on this platform, which is not allowed on the current versions.

In light of all these data, it seems more relevant to retain the SARS-CoV-2 positive result of these discordant samples on FilmArray RP2.1, regardless of the detection of one or both targets, which complies with the manufacturer's instructions. To our knowledge, this is the first study comparing FilmArray RP2.1 and MAScIR 2.0 assays for SARS-CoV-2 detection, both in Morocco and globally.

Moreover, our study highlights the urgent need for the standardization of RT-PCR assays and the importance of comparative studies between the numerous available tests and of taking part in external quality control programs.

## Figures and Tables

**Table 1:** Respiratory pathogen panel detected on FilmArray BioFire® RP2.1 plus [3].

Viruses	Bacteria
AdenovirusCoronavirus 229ECoronavirus HKU1Coronavirus NL63Coronavirus OC43Severe acute respiratory syndrome coronavirus 2 (SARS-CoV-2)Human metapneumovirusHuman rhinovirus/enterovirusInfluenza A, including subtypes H1, H3, and H1-2009Influenza BParainfluenza virus 1Parainfluenza virus 2Parainfluenza virus 3Parainfluenza virus 4Respiratory syncytial virus	*Bordetella parapertussisBordetella pertussisChlamydia pneumoniaeMycoplasma pneumoniae*

**Table 2:** Comparison of the results of FilmArray RP2.1 and MAScIR 2.0.

		MAScIR SARS-CoV-2 M kit 2.0	
		+	−
FilmArray BioFire® RP2.1 plus	+	**59**	**21**
	−	**0**	**80**

**Table 3:** Summary table of discrepant samples' results on each of the three assays.

No	FilmArray RP2.1			MAScIR 2.0			ARGENE			Other respiratory samples
	S	M	Other pathogens	S Ct	RdRp Ct	Result	N Ct	RdRp Ct	Result	
38	+	+	RSV	−	−	Ne	30	30	P	0
11	+	+	Metapneumovirus	−	−	Ne	33	32	P	0
81	+	+	Metapneumovirus	−	−	Ne	33	33	P	0
9	+	+	Rhinovirus/enterovirus + RSV	−	−	Ne	34	34	P	0
39	+	+	RSV	38	−	Ne	34	−	P	0
20	+	+	0	−	−	Ne	37	36	LP	0
82	+	+	0	−	−	Ne	−	−	Ne	D − 6: P on MAScIR
37	−	+	0	−	−	Ne	−	−	Ne	0
41	+	−	0	38	−	Ne	−	−	Ne	0
53	+	−	Rhinovirus/enterovirus + RSV	−	−	Ne	−	−	Ne	0
67	+	−	HCoV OC43 + PIV3	−	−	Ne	−	−	Ne	0
52	+	+	Rhinovirus/enterovirus + RSV	−	−	Ne	Not tested	0		
80	+	−	0	38	−	Ne	Not tested	0		
1	+	−	0	38	−	Ne	Not tested	0		
17	+	−	Metapneumovirus	−	−	Ne	Not tested	D − 5: Ne on MAScIR		
55	+	−	0	−	−	Ne	Not tested	D + 4: Ne on MAScIR		
69	+	−	0	−	−	Ne	Not tested	0		
73	+	−	Rhinovirus/enterovirus	−	−	Ne	Not tested	0		
74	+	−	0	38	−	Ne	Not tested	0		
36	−	+	Rhinovirus/enterovirus	−	−	Ne	Not tested	D − 1: LP on MAScIR		
79	−	+	HCoV OC43	−	−	Ne	Not tested	D + 2: Ne on MAScIR and FA (only HCoV OC43 detected)		

Ct: cycle threshold; D: collection day of samples included in our series; FA: filmArray RP2.1; HCoV: human coronavirus; LP: low positive; M: membrane gene; N: nucleocapsid gene; Ne: negative; P: positive; PIV3: parainfluenza virus 3; RdRp: RNA-dependent RNA polymerase gene; RSV: respiratory syncytial virus; S: spike gene; 0: none. Not tested: due to insufficient sample volume.

**Table 4:** Performance evaluation of FilmArray RP2.1 for SARS-CoV-2 detection in other series compared with our own [11–14].

Authors	Sample size		Comparative assay		FilmArray RP2.1 performance			
	Positive	Negative	NAAT	Targets	PPV [%]	NPV [%]	Sensitivity [%]	Specificity [%]
Eckbo et al. [11]	25	5	LDT	RdRp and E	100	100	100	100
Creager et al. [12]	50	50	Hologic panther fusion SARS-CoV-2 (15 samples)	ORF1ab	100	98	97.9	100
			LDT (15 samples)	N1 and N2				
			Roche cobas SARS-CoV-2 (20 samples)	ORF1a and E				
Johnson et al. [13]	16	17	GeneXpert xpert xpress SARS-CoV-2/Flu/RSV	E and N2	100	100	100	100
Jian et al. [14]	125 (50 wild-type SARS-CoV-2 specimens and 75 SARS-CoV-2 alpha variant specimens)	200	LDT	ORF1ab and E	100	99.5	99.2	100
Tazi et al. (our series)	80	80	MAScIR SARS-CoV-2 M kit 2.0	RdRp and S	73.8	100	100	79.2

E: envelope gene; LDT: laboratory-developed test; N: nucleocapsid gene; NPV: negative predictive value; ORF: open reading frame; PPV: positive predictive value; RdRp: RNA-dependent RNA polymerase gene; S: spike gene.

## Data Availability

The data used to support the findings of this study have been provided by the authors during the submission of the article.

## Supplementary Materials

Supplementary Materials The data collected in our study were provided in two tables: the first table contains the data of SARS-CoV-2 positive samples on FilmArray (i.e., patients' age, sex, and symptomatology, and the results of chest CT-scan and nonspecific biological tests, as well as the results of the samples on each of the three PCR assays compared in our study, and those of subsequent or previous samples collected from these patients), whereas the second table contains the data of SARS-CoV-2 negative samples on FilmArray.
