# Supplementary material for: Comparative Performance Evaluation of FilmArray BioFire RP2.1 and MAScIR 2.0 Assays for SARS-CoV-2 Detection
Source: Adv Virol. 2022 Jun 1;2022:4510900. doi: 10.1155/2022/4510900 (PMC9177333; doi:10.1155/2022/4510900)
Supplement: Supplementary Materials — The data collected in our study were provided in two tables: the first table contains the data of SARS-CoV-2 positive samples on FilmArray (i.e., patients' age, sex, and symptomatology, and the results of chest CT-scan and nonspecific biological tests, as well as the results of the samples on each of the three PCR assays compared in our study, and those of subsequent or previous samples collected from these patients), whereas the second table contains the data of SARS-CoV-2 negative samples on FilmArray. [file 4510900.f1.zip › 4510900.f1/Supplementary File - Comparative performance evaluation of FilmArray and MAScIR assays for SARS-CoV-2 detection - Abbreviations.pdf]

Ct : Cycle threshold
D : Collection day of samples included in our series
DSO : Days from Symptom Onset
FA : FilmArray RP2.1
HCoV: Human Coronavirus
IC : Internal Control
LP: Low positive
M: Membrane gene
mo : months old
N: Nucleocapsid gene
NA : Not available
Ne: Negative
NQC : National Quality Control
NT : Not Tested
P: Positive
PIV : Parainfluenza virus
RdRp : RNA-dependent RNA polymerase gene
RSV: Respiratory Syncytial Virus
S: Spike gene
yo : years old
WBC : White Blood Cells
0 : Absent
1 : Present

Ct : Cycle threshold
M : Membrane gene
mo : months old
Ne : Negative
PIV : Parainfluenza virus
RdRp : RNA-dependent RNA polymerase gene
RSV: Respiratory Syncytial Virus
S: Spike gene
yo : years old
0 : None

Samples N°26, 34, 35, 40 and 49 : were the five positive samples on FA excluded from our series, due to insufficient volume for comparative testing on MAScIR 2.0
