# Supplementary material for: Comparative Performance Evaluation of FilmArray BioFire RP2.1 and MAScIR 2.0 Assays for SARS-CoV-2 Detection
Source: Adv Virol. 2022 Jun 1;2022:4510900. doi: 10.1155/2022/4510900 (PMC9177333; doi:10.1155/2022/4510900)
Supplement: Supplementary Materials — The data collected in our study were provided in two tables: the first table contains the data of SARS-CoV-2 positive samples on FilmArray (i.e., patients' age, sex, and symptomatology, and the results of chest CT-scan and nonspecific biological tests, as well as the results of the samples on each of the three PCR assays compared in our study, and those of subsequent or previous samples collected from these patients), whereas the second table contains the data of SARS-CoV-2 negative samples on FilmArray. [file 4510900.f1.zip › 4510900.f1/Supplementary File - Comparative performance evaluation of FilmArray and MAScIR assays for SARS-CoV-2 detection - Negative Samples.pdf]

N°	Age	Sex	FilmArray RP2.1			MAScIR 2.0		
			S	M	Other Pathogens	S Ct	RdRp Ct	Result
N1	47 yo	M	-	-	PIV4	-	-	**Ne**
N2	56 yo	M	-	-	0	-	-	**Ne**
N3	76 yo	F	-	-	0	-	-	**Ne**
N4	8 mo	M	-	-	PIV3 + PIV4 + Rhinovirus/Enterovirus	-	-	**Ne**
N5	2 yo	M	-	-	0	-	-	**Ne**
N6	22 yo	F	-	-	0	-	-	**Ne**
N7	84 yo	M	-	-	0	-	-	**Ne**
N8	81 yo	F	-	-	0	-	-	**Ne**
N9	2 yo	F	-	-	PIV3	-	-	**Ne**
N10	14 yo	F	-	-	0	-	-	**Ne**
N11	6 mo	F	-	-	PIV4 + Rhinovirus/Enterovirus	-	-	**Ne**
N12	1 yo	M	-	-	0	-	-	**Ne**
N13	1 yo	F	-	-	0	-	-	**Ne**
N14	1 day	M	-	-	RSV + Rhinovirus/Enterovirus	-	-	**Ne**
N15	7 mo	M	-	-	PIV3	-	-	**Ne**
N16	59 yo	F	-	-	0	-	-	**Ne**
N17	2 yo	M	-	-	RSV + PIV3	-	-	**Ne**
N18	4 mo	F	-	-	RSV + PIV3	-	-	**Ne**
N19	3 yo	M	-	-	PIV3	-	-	**Ne**
N20	61 yo	M	-	-	0	-	-	**Ne**
N21	2 days	F	-	-	RSV	-	-	**Ne**
N22	1 day	F	-	-	RSV	-	-	**Ne**
N23	1 mo	F	-	-	RSV	-	-	**Ne**
N24	5 mo	M	-	-	RSV	-	-	**Ne**
N25	3 days	M	-	-	Adenovirus + RSV + Metapneumovirus	-	-	**Ne**
N26	18 mo	F	-	-	RSV	-	-	**Ne**
N27	18 mo	F	-	-	RSV + Rhinovirus/Enterovirus	-	-	**Ne**
N28	18 mo	M	-	-	RSV	-	-	**Ne**
N29	9 mo	F	-	-	Rhinovirus/Enterovirus	-	-	**Ne**
N30	16 mo	F	-	-	Metapneumovirus + Rhinovirus/Enterovirus	-	-	**Ne**
N31	2 yo	M	-	-	RSV	-	-	**Ne**
N32	2 mo	M	-	-	Rhinovirus/Enterovirus	-	-	**Ne**
N33	18 mo	M	-	-	Rhinovirus/Enterovirus	-	-	**Ne**
N34	9 mo	M	-	-	RSV + Rhinovirus/Enterovirus	-	-	**Ne**
N35	15 yo	M	-	-	Rhinovirus/Enterovirus	-	-	**Ne**
N36	3 yo	F	-	-	Rhinovirus/Enterovirus	-	-	**Ne**
N37	2 yo	M	-	-	RSV	-	-	**Ne**
N38	16 mo	M	-	-	Rhinovirus/Enterovirus	-	-	**Ne**
N39	4 yo	F	-	-	Metapneumovirus	-	-	**Ne**
N40	1 day	F	-	-	PIV3 + RSV	-	-	**Ne**
N41	1 mo	M	-	-	RSV + Rhinovirus/Enterovirus	-	-	**Ne**
N42	4 yo	M	-	-	RSV	-	-	**Ne**
N43	10 mo	F	-	-	RSV	-	-	**Ne**
N44	7 yo	M	-	-	Rhinovirus/Enterovirus	-	-	**Ne**
N45	2 yo	M	-	-	RSV	-	-	**Ne**
N46	4 yo	M	-	-	Rhinovirus/Enterovirus	-	-	**Ne**
N47	41 yo	F	-	-	0	-	-	**Ne**
N48	1 mo	F	-	-	RSV	-	-	**Ne**
N49	18 mo	F	-	-	RSV	-	-	**Ne**
N50	18 mo	M	-	-	RSV + Rhinovirus/Enterovirus	-	-	**Ne**
N51	14 mo	M	-	-	RSV	-	-	**Ne**
N52	4 mo	M	-	-	PIV3 + RSV	-	-	**Ne**
N53	13 yo	M	-	-	Rhinovirus/Enterovirus	-	-	**Ne**
N54	1 day	M	-	-	RSV	-	-	**Ne**
N55	18 mo	M	-	-	Rhinovirus/Enterovirus	-	-	**Ne**
N56	8 mo	F	-	-	Rhinovirus/Enterovirus	-	-	**Ne**
N57	61 yo	M	-	-	0	-	-	**Ne**
N58	3 days	M	-	-	Rhinovirus/Enterovirus	-	-	**Ne**
N59	1 day	F	-	-	Rhinovirus/Enterovirus	-	-	**Ne**
N60	4 yo	M	-	-	Adenovirus + Rhinovirus/Enterovirus	-	-	**Ne**
N61	21 mo	M	-	-	RSV	-	-	**Ne**
N62	3 yo	F	-	-	Rhinovirus/Enterovirus	-	-	**Ne**
N63	8 mo	M	-	-	RSV	-	-	**Ne**
N64	5 yo	M	-	-	Rhinovirus/Enterovirus	-	-	**Ne**
N65	2 yo	F	-	-	Rhinovirus/Enterovirus	-	-	**Ne**
N66	2 yo	M	-	-	RSV + Rhinovirus/Enterovirus	-	-	**Ne**
N67	1 yo	M	-	-	RSV + Rhinovirus/Enterovirus	-	-	**Ne**
N68	2 yo	F	-	-	RSV + Rhinovirus/Enterovirus	-	-	**Ne**
N69	8 mo	M	-	-	Rhinovirus/Enterovirus	-	-	**Ne**
N70	2 yo	F	-	-	0	-	-	**Ne**
N71	63 yo	M	-	-	Rhinovirus/Enterovirus	-	-	**Ne**
N72	1 day	M	-	-	Metapneumovirus	-	-	**Ne**
N73	8 mo	M	-	-	RSV	-	-	**Ne**
N74	1 mo	M	-	-	RSV + Rhinovirus/Enterovirus	-	-	**Ne**
N75	3 yo	F	-	-	Rhinovirus/Enterovirus	-	-	**Ne**
N76	8 mo	M	-	-	RSV	-	-	**Ne**
N77	5 mo	M	-	-	RSV	-	-	**Ne**
N78	2 mo	M	-	-	RSV	-	-	**Ne**
N79	7 yo	M	-	-	RSV	-	-	**Ne**
N80	26 yo	F	-	-	Rhinovirus/Enterovirus	-	-	**Ne**
