# Supplementary material for: Comparative Performance Evaluation of FilmArray BioFire RP2.1 and MAScIR 2.0 Assays for SARS-CoV-2 Detection
Source: Adv Virol. 2022 Jun 1;2022:4510900. doi: 10.1155/2022/4510900 (PMC9177333; doi:10.1155/2022/4510900)
Supplement: Supplementary Materials — The data collected in our study were provided in two tables: the first table contains the data of SARS-CoV-2 positive samples on FilmArray (i.e., patients' age, sex, and symptomatology, and the results of chest CT-scan and nonspecific biological tests, as well as the results of the samples on each of the three PCR assays compared in our study, and those of subsequent or previous samples collected from these patients), whereas the second table contains the data of SARS-CoV-2 negative samples on FilmArray. [file Supplementary File - Comparative performance evaluation of FilmArray and MAScIR assays for SARS-CoV-2 detection - Positive Samples.pdf › 4510900.f1/Supplementary File - Comparative performance evaluation of FilmArray and MAScIR assays for SARS-CoV-2 detection - Positive Samples.pdf]

N°	Age	Sex	DSO	Symptoms											FilmArray RP2.1			MAScIR 2.0				ARGENE				Anterior respiratory samples	Ulterior respiratory samples	Thoracic CT Scan	Non specific biology		
				Fever	Cough	Dyspnea	Sore throat	Respiratory distress	Headache	Myalgia	Diarrhea	Abdominal pain	Vomiting	Other symptoms	S	M	Other pathogens	IC Ct	S Ct	RdRp Ct	Result	IC Ct	N Ct	RdRp Ct	Result				C-Reactive Protein	Procalcitonin	Haemogram
1	18 mo	M	NA	NA	NA	1	NA	1	NA	NA	NA	NA	NA		+	-	0	+	38	-	Ne				Insufficient volume	0	0	NA	NA	NA	NA
2	74 yo	M	11	1	0	1	0	0	1	1	NA	NA	NA		+	+	0	+	27	25	P				NT	0	0	NA	NA	NA	NA
3	13 mo	M	NA	1	1	1	0	0	0	0	0	0	0		+	+	0	24	34	34	LP				NT	0	0	NA	NA	NA	NA
4	76 yo	M	11	1	1	1	NA	NA	NA	NA	NA	NA	NA		+	+	0	25	22	21	P				NT	D-1 : Sample n°5	0	NA	NA	NA	NA
5	76 yo	M	10	1	1	1	NA	NA	NA	NA	NA	NA	NA		+	+	0	27	26	24	P				NT	0	D+1 : Sample n°4	NA	NA	NA	NA
6	79 yo	M	3	0	1	1	NA	NA	NA	NA	NA	NA	NA		+	+	0	25	24	23	P				NT	0	0	NA	NA	NA	NA
7	85 yo	F	2	1	1	1	0	0	0	1	NA	NA	NA		+	+	0	34,9	25	23	P				NT	0	0	Aspect of COVID-19 pneumonia	NA	NA	NA
8	86 yo	M	5	0	0	1	NA	NA	NA	NA	NA	NA	NA		+	+	0	23	21	20	P				NT	0	0	NA	NA	NA	NA
9	9 mo	M	NA	NA	NA	1	NA	1	NA	NA	NA	NA	NA		+	+	Rhinovirus/Enterovirus + RSV	+	-	-	Ne	27	34	34	P	0	0	NA	NA	NA	NA
10	57 yo	F	20	0	0	0	NA	NA	NA	NA	NA	NA	NA		-	+	0	+	-	36	LP				NT	0	0	NA	NA	NA	NA
11	18 mo	M	5	1	1	1	0	1	0	0	0	0	0		+	+	Metapneumovirus	+	-	-	Ne	28	33	32	P	0	0	NA	9 mg/L	NA	WBC : 11 G/L ; Neutrophils : 4 G/L ; Lymphocytes : 6,6 G/L
12	4 mo	F	5	1	1	1	0	0	0	0	0	0	0		-	+	RSV	+	-	36.3	LP				NT	0	D+2 : Ne on FA (only RSV detected)	NA	NA	NA	WBC : 19 G/L ; Neutrophils : 6 G/L ; Lymphocytes : 11 G/L
13	31 yo	F	7	1	0	1	NA	NA	NA	NA	NA	NA	NA		+	+	Rhinovirus/Enterovirus	24	30	29	P				NT	0	0	NA	NA	NA	NA
14	2 yo	M	0	1	1	1	0	1	0	0	0	0	0		+	+	0	+	28	30	P				NT	0	D+9 : Ne on MAScIR	NA	NA	NA	NA
15	9 yo	F	3	1	0	1	0	0	0	0	0	0	0		+	+	0	+	19	19	P				NT	0	D+8 : LP on MAScIR, D+12 : Ne on MAScIR	NA	NA	NA	NA
16	40 days	F	1	1	1	1	0	1	0	0	Unspecified digestive symptoms				+	+	RSV	26	36	35	LP				NT	0	D+6 : Ne on FA (only RSV detected)	NA	NA	NA	NA
17	61 yo	M	2	NA	NA	NA	NA	NA	NA	NA	NA	NA	NA		+	-	Metapneumovirus	+	-	-	Ne				Insufficient volume	D-5 : Ne on MAScIR	0	NA	NA	NA	NA
18	51 yo	F	2	NA	NA	NA	NA	NA	NA	NA	NA	NA	NA		+	+	0	25	34	32	LP				NT	0	0	NA	NA	NA	NA
19	56 yo	F	3	NA	NA	NA	NA	NA	NA	NA	NA	NA	NA		+	+	0	24	35	-	LP				NT	D-10 : Ne on FA	0	NA	NA	NA	NA
20	48 yo	M	NA	0	0	0	0	0	0	0	0	0	0		+	+	0	+	-	-	Ne	26	37	36	LP	0	0	NA	NA	NA	NA
21	33 yo	M	19	NA	NA	NA	NA	NA	NA	NA	NA	NA	NA	Sepsis	+	+	0	22	21	20	P				NT	D-12 : Sample n°25	0	NA	NA	NA	NA
22	36 yo	M	NA	NA	NA	NA	NA	NA	NA	NA	NA	NA	NA		+	+	0	24	33	33	LP				NT	0	0	NA	NA	NA	NA
23	11 mo	M	NA	0	0	0	1	0	0	0	NA	NA	NA	Rhinorrhea	+	+	RSV	25	20	19	P				NT	0	D+8 : P on MAScIR	NA	NA	NA	NA
24	63 yo	M	4	1	0	1	0	1	0	0	0	0	0		+	+	0	25	25	24	P				NT	0	0	NA	NA	NA	NA
25	33 yo	M	7	1	0	0	NA	0	NA	NA	NA	NA	NA		+	+	0	22	25	24	P				NT	0	D+12 : Sample n°21	NA	NA	NA	NA
27	69 yo	M	2	1	1	1	0	0	0	0	0	0	1		+	+	0	28	16	16	P				NT	0	0	NA	NA	NA	NA
28	86 yo	M	NA	NA	NA	NA	NA	NA	NA	NA	NA	NA	NA		+	+	0	24	29	28	P				NT	0	0	NA	NA	NA	NA
29	1 mo	M	1	0	1	1	0	1	0	0	NA	NA	NA		+	+	0	25	23	22	P				NT	0	0	NA	NA	NA	NA
30	1 mo	F	4	1	1	1	0	1	0	0	NA	NA	NA		+	+	0	+	20	22	P				NT	0	0	NA	NA	NA	NA
31	5 mo	F	NA	1	1	1	0	1	0	0	NA	NA	NA		+	+	0	+	20	22	P				NT	0	D+9 : Ne on MAScIR	CO-RADS 4 lesions (10% of pulmonary involvement)	NA	NA	NA
32	43 yo	M	1	1	0	0	NA	NA	NA	NA	NA	NA	NA		+	+	0	23	35	32	LP				NT	0	0	NA	NA	NA	NA
33	84 yo	F	2	1	1	1	NA	NA	NA	NA	NA	NA	NA		+	+	Adenovirus	23	19	19	P				NT	0	0	NA	NA	NA	NA
36	3 yo	M	6	1	0	1	0	1	0	0	NA	NA	1		-	+	Rhinovirus/Enterovirus	+	-	-	Ne				Insufficient volume	D-1 : LP on MAScIR	0	Not realized	14 mg/L	NA	WBC : 17 G/L ; Neutrophils : 13 G/L ; Lymphocytes : 3 G/L
37	79 yo	F	2	1	1	1	0	1	0	0	0	0	0		-	+	0	+	-	-	Ne	26	-	-	Ne	0	0	NA	NA	NA	NA
38	61 yo	F	3	1	1	1	NA	NA	NA	NA	NA	NA	NA		+	+	RSV	+	-	-	Ne	26	30	30	P	0	0	NA	NA	NA	NA
39	60 yo	M	2	NA	NA	NA	NA	NA	NA	NA	NA	NA	NA		+	+	RSV	+	38	-	Ne	27	34	-	P	0	0	NA	NA	NA	NA
41	27 yo	F	29	NA	NA	NA	NA	NA	NA	NA	NA	NA	NA		+	-	0	+	38	-	Ne	27	-	-	Ne	0	0	NA	NA	NA	NA
42	74 yo	F	7	0	0	1	0	1	0	0	0	0	0		+	+	0	28	17	19	P				NT	0	0	NA	NA	NA	NA
43	80 yo	F	NA	1	1	1	0	1	1	0	0	0	0		+	+	0	25	25	25	P				NT	0	0	NA	NA	NA	NA
44	54 yo	M	5	1	0	0	NA	NA	NA	NA	NA	NA	NA		+	+	0	25	37	-	LP				NT	0	0	NA	NA	NA	NA
45	65 yo	F	5	0	0	1	NA	NA	NA	NA	NA	NA	NA		+	+	0	26	38	35	LP				NT	0	0	NA	NA	NA	NA
46	43 yo	F	NA	NA	NA	NA	NA	NA	NA	NA	NA	NA	NA		+	+	0	26	37	34	LP				NT	0	0	NA	NA	NA	NA
47	63 yo	F	3	1	1	1	1	0	0	0	0	0	0	Anosmia + agueusia	+	+	0	28	30	29	P				NT	0	0	NA	NA	NA	NA
48	14 mo	F	4	1	1	0	0	0	0	0	0	0	0		+	+	0	28	20	19	P				NT	0	D+8 : P on MAScIR ; D+12 : P on MAScIR ; D+16 : LP on MAScIR ; D+22 : Ne on MAScIR	NA	NA	NA	NA
50	81 yo	M	NA	1	1	1	NA	1	NA	NA	NA	NA	NA		+	+	0	27	26	25	P				NT	0	D+2 : P on MAScIR	NA	NA	NA	NA
51	13 yo	M	NA	NA	NA	NA	NA	NA	NA	NA	NA	NA	NA		+	+	0	25	27	27	P				NT	0	0	NA	NA	NA	NA
52	5 yo	F	1	0	1	1	0	0	0	0	0	0	0		+	+	Rhinovirus/Enterovirus + RSV	+	-	-	Ne				Insufficient volume	0	0	NA	NA	NA	NA
53	8 mo	M	NA	1	1	1	0	0	0	0	0	0	0		+	-	Rhinovirus/Enterovirus + RSV	+	-	-	Ne	27	-	-	Ne	0	0	NA	NA	NA	NA
54	68 yo	F	NA	NA	NA	NA	NA	NA	NA	NA	NA	NA	NA		+	+	0	+	28	30	P				NT	0	D+6 : LP on MAScIR ; D+12 : Ne on MAScIR	NA	NA	NA	NA
55	40 yo	F	3	1	0	1	NA	NA	NA	NA	NA	NA	NA		+	-	0	+	-	-	Ne				Insufficient volume	0	D+4 : Ne on MAScIR	NA	NA	NA	NA
56	67 yo	M	NA	1	1	1	0	0	1	0	0	0	0		+	+	0	+	20	20	P				NT	0	0	NA	NA	NA	NA
57	30 yo	F	16	1	1	0	0	0	1	1	1	1	1		+	+	0	+	36.9	-	LP				NT	0	0	NA	NA	NA	NA
58	60 yo	M	5	1	0	1	NA	NA	NA	NA	NA	NA	NA		-	+	Rhinovirus/Enterovirus	+	-	36	LP				NT	0	0	NA	NA	NA	NA
59	68 yo	M	NA	NA	NA	NA	NA	NA	NA	NA	NA	NA	NA		+	+	0	+	28	28	P				NT	0	D+5 : Sample n°60 ; D+18 : Ne on MAScIR	NA	NA	NA	NA
60	68 yo	M	NA	NA	NA	NA	NA	NA	NA	NA	NA	NA	NA		-	+	0	+	38	36.7	LP				NT	D-5 : Sample n°59	D+13 : Ne on MAScIR	NA	NA	NA	NA
61	71 yo	F	7	1	1	1	NA	1	NA	NA	NA	NA	NA		+	+	0	+	17	17	P				NT	Same Day : Sample n°62	0	NA	NA	NA	NA
62	71 yo	F	7	1	1	1	NA	1	NA	NA	NA	NA	NA		+	+	0	+	17	17	P				NT	Sample Day : Sample n°61		NA	NA	NA	NA
63	85 yo	F	5	1	1	1	0	0	0	0	0	0	0		+	+	0	+	21	20	P				NT	0	D+7 : P on MAScIR ; D+10 : P on MAScIR ; D+14 : P on MAScIR ; D+25 : Ne on MAScIR	NA	NA	NA	NA
64	73 yo	F	3	1	1	1	NA	1	NA	NA	NA	NA	NA		+	+	0	25	16	16	P				NT	0	0	NA	NA	NA	NA
65	65 yo	M	NA	1	1	0	0	0	0	1	0	1	1		+	+	0	26	29	28	P				NT	0	D+14 : Ne on MAScIR	NA	NA	NA	NA
66	72 yo	M	7	1	1	1	0	1	0	0	0	0	0		+	+	0	+	13	12	P				NT	0	D+7 : P on MAScIR	CO-RADS 5 lesions (50% of pulmonary involvement)	198 mg/L	NA	WBC : Normal (value NA) ; Neutrophils : Normal (value NA) ; Lymphocytes : 90 G/L
67	58 yo	M	5	NA	NA	NA	NA	NA	NA	NA	NA	NA	NA		+	-	HCoV OC43 + PIV3	+	-	-	Ne	29	-	-	Ne	0	0	NA	NA	NA	NA
68	56 yo	F	9	1	1	1	1	0	0	0	0	0	0		+	+	0	29	23	22	P				NT	0	0	NA	NA	NA	NA
69	48 yo	M	NA	NA	NA	1	NA	1	NA	NA	NA	NA	NA		+	-	0	+	-	-	Ne				Insufficient volume	0	0	NA	NA	NA	NA
70	30 yo	F	NA	NA	NA	NA	NA	NA	NA	NA	NA	NA	NA		+	+	0	31	19	17	P				NT	0	0	NA	NA	NA	NA
71	37 yo	M	NA	0	0	0	0	0	0	0	0	0	0		+	+	0	28	24	22	P				NT	0	0	NA	NA	NA	NA
72	63 yo	M	16	1	0	1	0	1	1	1	0	0	0		+	+	0	+	14	15	P				NT	0	0	CO-RADS 5 lesions (50% of pulmonary involvement)	NA	NA	NA
73	27 yo	F	NA	NA	NA	NA	NA	NA	NA	NA	NA	NA	NA	Etiological diagnosis of diabetic ketoacidosis	+	-	Rhinovirus/Enterovirus	+	-	-	Ne				Insufficient volume	0	0	NA	NA	NA	NA
74	18 yo	F	1	NA	NA	NA	NA	NA	NA	NA	NA	NA	NA	Etiological diagnosis of diabetic ketoacidosis	+	-	0	+	38	-	Ne				Insufficient volume	0	0	Not realized	Normal (value NA)	Normal (value NA)	WBC : 23 G/L ; Neutrophils : 18 G/L
75	74 yo	F	NA	NA	NA	NA	NA	NA	NA	NA	NA	NA	NA		+	+	0	+	27	25	P				NT	0	D+8 : LP on MAScir	NA	NA	NA	NA
76	70 yo	M	9	0	0	1	NA	1	NA	NA	NA	NA	NA		+	+	0	+	29	28	P				NT	0	0	CO-RADS 5 lesions	NA	NA	NA
77	NQC	NQC	NQC	NQC	NQC	NQC	NQC	NQC	NQC	NQC	NQC	NQC	NQC		+	+	0	+	33	33	LP				NT	0	0	NQC	NQC	NQC	NQC
78	73 yo	M	6	1	0	1	1	1	0	0	0	0	1		+	+	0	+	33	32	LP				NT	Same Day : Ne on MAScIR	D+5 : Ne on MAScIR	Bilateral segmental pulmonary embolism	7,48 mg/L	NA	WBC : 14 G/L ; Neutrophils : 10 G/L ; Lymphocytes : 3 G/L
79	64 yo	F	6	0	1	1	1	0	0	0	0	0	0		-	+	HCoV OC43	+	-	-	Ne				Insufficient volume	0	D+2 : Ne on MAScIR and FA (only HCoV OC43 detected)	NA	NA	NA	NA
80	78 yo	M	3	1	1	1	1	1	0	0	0	0	0		+	-	0	+	38	-	Ne				Insufficient volume	0	0	Aspect of COVID-19 penumonia (10% of pulmonary involvement)	NA	NA	NA
81	1 yo	F	NA	1	0	1	0	1	0	0	0	0	0		+	+	Metapneumovirus	+	-	-	Ne	28	33	33	P	0	0	NA	NA	NA	NA
82	71 yo	F	10	1	1	1	NA	NA	NA	NA	NA	NA	NA		+	+	0	+	-	-	Ne	27	-	-	Ne	D-6 : P on MAScIR	0	NA	NA	NA	NA
83	49 yo	M	9	0	0	1	NA	NA	NA	NA	NA	NA	NA		+	+	0	+	28	26	P				NT	0	0	NA	NA	NA	NA
84	3 mo	F	NA	1	1	1	0	0	0	0	NA	NA	NA		+	+	RSV	+	35	34	LP				NT	0	0	NA	NA	NA	NA
85	27 yo	F	NA	1	NA	1	NA	NA	NA	NA	NA	NA	NA		+	+	0	+	16	19	P				NT	0	0	NA	NA	NA	NA
